# Supplementary material for: Causal Relationship between Aging and Anorexia Nervosa: A White-Matter-Microstructure-Mediated Mendelian Randomization Analysis
Source: Biomedicines. 2024 Aug 16;12(8):1874. doi: 10.3390/biomedicines12081874 (PMC11351342; doi:10.3390/biomedicines12081874)
Supplement: Supplementary file 1 [file biomedicines-12-01874-s001.zip › Supplementary Figure/Figure S9/Leaveoneout plot of MR analysis for ubm-b-1604 to AN.pdf]

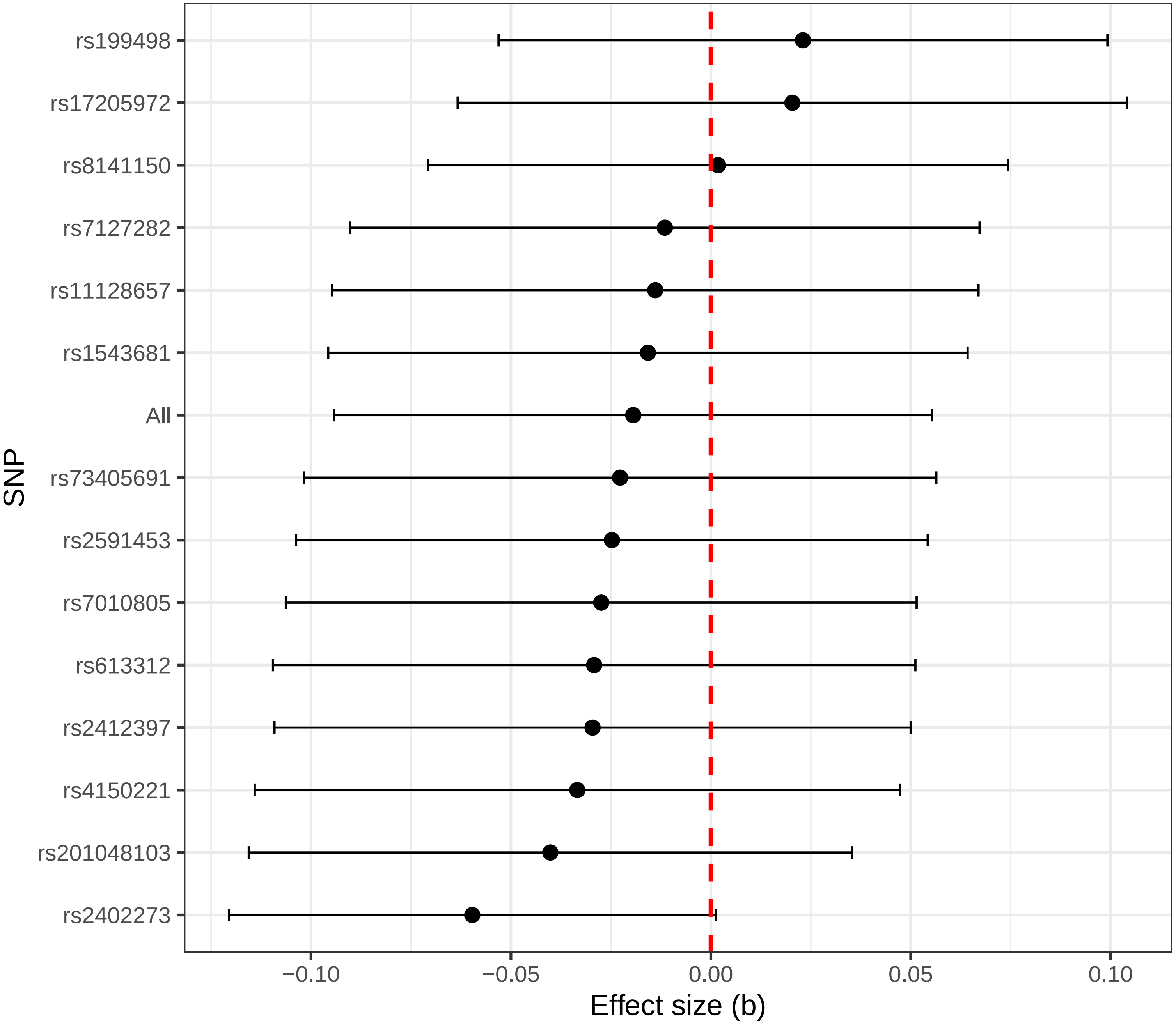

rs199498
rs17205972
rs8141150
rs7127282
rs11128657
rs1543681
All
rs73405691
rs2591453
rs7010805
rs613312
rs2412397
rs4150221
rs201048103
rs2402273
SNP
−0.10
−0.05
0.00
0.05
0.10
Effect size (b)
